# Comparative genomic analyses of four novel *Ramlibacter* species and the cellulose-degrading properties of *Ramlibacter cellulosilyticus* sp. nov.

**DOI:** 10.1038/s41598-022-25718-w

**Published:** 2022-12-08

**Authors:** Minchung Kang, Geeta Chhetri, Inhyup Kim, Yoonseop So, Taegun Seo

**Affiliations:** Department of Life Science, Dongguk University-Seoul, Goyang, 10326 Korea

**Keywords:** Microbiology, Microbial ecology

## Abstract

In this study, four novel bacterial strains, USB13^T^, AW1^T^, GTP1^T^, and HM2^T^, were isolated from various environments in Busan and Jeju Island, Republic of Korea. The 16S rRNA sequencing results indicated that the four novel strains belong to the genus *Ramlibacter.* All four strains were tested for their potential cellulolytic properties, where strain USB13^T^ was identified as the only novel bacterium and the first within its genus to show cellulolytic activity. When tested, the highest activities of endoglucanase, exoglucanase, *β*-glucosidase, and filter paper cellulase (FPCase) were 1.91 IU/mL, 1.77 IU/mL, 0.76 IU/mL, and 1.12 IU/mL, respectively at pH 6.0. Comparisons of draft whole genome sequences (WGS) were also made using average nucleotide identity, digital DNA-DNA hybridization values, and average amino acid identity values, while whole genome comparison was visualized using the BLAST Ring Image Generator. The G + C contents of the strains ranged from 67.9 to 69.9%, while genome sizes ranged from 4.31 to 6.15 Mbp. Based on polyphasic evidence, the novel strains represent four new species within the genus *Ramlibacter*, for which the names *Ramlibacter cellulosilyticus* sp. nov. (type strain, USB13^T^ = KACC 21656^T^ = NBRC 114839^T^) *Ramlibacter aurantiacus* sp. nov. (type strain, AW1^T^ = KACC 21544^T^ = NBRC 114862^T^), *Ramlibacter albus* sp. nov. (type strain, GTP1^T^ = KACC 21702^T^ = NBRC 114488^T^), and *Ramlibacter pallidus* sp. nov. (type strain, HM2^T^ = KCTC 82557^T^ = NBRC 114489^T^) are proposed.

## Introduction

The genus *Ramlibacter*, which belongs to the family *Comamonadaceae*, within the order *Burkholderiales*, and class *Betaproteobacteria*, was first proposed in 2003 by Heulin et al. with *Ramlibacter tataouinensis* TTB310^T^ introduced as its type species^1^. As of this writing, a total of 11 species have been validly published according to the List of Prokaryotic names with Standing in Nomenclature (LPSN; https://lpsn.dsmz.de/genus/ramlibacter). ^2^ These species have been isolated from various soils including subdesert soil^1^, ginseng soil^3,4^, rhizosphere soil^5^, forest soil^6–8^, and garden soil^9^. Members of the genus *Ramlibacter* are Gram-negative, asporogenous, and rod- or coccoid-shaped bacteria. The DNA G + C content of members of the genus ranges from 68.5% to 70.0%. Additionally, they are known to have ubiquinone-8 (Q-8) as their major respiratory quinone, as well as palmitic acid (C_16:0_), summed feature 3 (consisting of C_16:1_ ω7c and/or C_16:1_ ω6c), and summed feature 8 (consisting of C_18:1_ ω7c and/or C_18:1_ ω6c) as their major fatty acids. Currently, there have not been any reports of *Ramlibacter* species with cellulolytic activity, making *Ramlibacter cellylosilyticus* sp. nov. the first within the genus.

Cellulose is a homopolymer of D-glucose units linked by *β*-1,4-glycosidic bonds. Despite being Earth’s most abundant biomass component, its recalcitrant nature has made cellulose one of the least utilized bioresources^10^. Generally, cellulose is converted to glucose either chemically or enzymatically. Inorganic acids are used during chemical hydrolysis, resulting in degradation products that are toxic to microbes which are used in following fermentation steps. Thus, detoxification steps are necessary in order to remove the harmful byproducts. Opposed to chemical hydrolysis, enzymatic hydrolysis does not require these additional detoxifications steps, making enzymatic hydrolysis an eco-friendlier method to obtain glucose^11^. Hydrolysis of cellulose commonly requires three enzymes to degrade it into glucose monomers: endoglucanases (endo-1,4-*β*-endoglucanase), exoglucanases (exo-1,4-*β*-endoglucanase), and *β*-glucosidases^12^. Endoglucanases cut random amorphous sites within the cellulose polymer, generating cellulose ends that exoglucanases in turn act on to cleave from the end. This results in producing products such as tetrasaccharides or cellobiose, a disaccharide, which is then further hydrolyzed by *β*-glucosidases to generate glucose^13^. Additionally, the synergistic action of multiple carbohydrate-active enzymes (CAZymes) such as glycosyl hydrolases, also known as glycoside hydrolases (GH), is required to degrade cellulose^14^. While the complete mechanics are not certain, the main three cellulase enzymes and CAZymes work together through coordinated and complementary oxidative, hydrolytic, and non-hydrolytic activities, which in turn contribute to the total cellulase activity^15^.

Due to the abundance of cellulosic biomass and its potential as biofuel, there is currently an unprecedented demand for cellulolytic microbes. Although fungi were once thought to be the only viable solution for cellulose degradation, the focus has now shifted to cellulolytic bacteria due to their faster growth rates and ability to withstand extreme environments^10,17^. Some examples are *Fibrobacter*^16,17^, *Ruminococcus*^18^, and *Cellulomonas*^19^, where many species have been isolated from the rumen, intestinal tract, and feces of herbivores.

In this study, we present the chemotaxonomic, physiological, and genomic comparisons between the cellulolytic strain USB13^T^, and non-cellulolytic strains AW1^T^, GTP1^T^, and HM2^T^. Additionally, cellulolytic activity was assessed by measuring endoglucanase, exoglucanase, and *β*-glucosidase activity, as well as total cellulase activity, which was assessed by measuring the activity of filter paper hydrolysis.

## Materials and methods

### Sample sites, isolation, and maintenance

During an expedition investigating indigenous bacterial communities in South Korea, strain USB13^T^ was isolated from a sea water sample collected from a shallow coastal region in Haeundae Beach, Busan (35° 09′ 29.7′′ N, 129° 09′ 35.4′′ E). Strains AW1^T^, GTP1^T^, and HM2^T^ were isolated from soil samples collected, respectively in the Jeju Island regions of Aewol (33° 28′ 15.8′′ N, 126° 26′ 20.7′′ E), Seogwipo (33° 17′ 37.8′′ N, 126° 25′ 15.6′′ E), and Seopjikoji Beach (33° 25′ 26.8′′ N, 126° 55′ 51.2′′ E). The sea water sample was subsequently diluted with a sterile 0.85% (w/v) NaCl solution using the standard ten-fold serial dilution method (10^−1^ to 10^−4^). The diluted sample was spread onto Reasoner’s 2A agar (R2A; MB cell) and incubated for 5 days at 28 °C. Soil samples were submerged in 25 mL of sterile 0.85% (w/v) NaCl solution and rotated for 1 h by using an orbital shaker. The supernatant was then diluted using the standard ten-fold serial dilution method (10^−1^ to 10^−4^) and spread onto R2A agar, which was incubated for 5 days at 28 °C. Colonies were repeatedly selected and subcultured onto new R2A agar plates to ensure the purity of the strain. The purified colonies were then sent to Solgent Co., Ltd. (Daejeon, South Korea) for 16S rRNA gene sequence analysis. The novel strains were designated USB13^T^, AW1^T^, GTP1^T^, and HM2^T^ and were stored at −80 °C in R2A broth containing 50% glycerol (w/v) for future experiments. For strain conservation, the four novel strains were deposited in the Korean Agricultural Culture Collection (KACC; Jeonju, South Korea), the Korean Collection for Type Cultures (KCTC; Jeongeup, South Korea), and the NITE Biological Resource Center (NBRC; Shibuya, Japan).

### Chemotaxonomic characterization

For chemotaxonomic characterization, cells of the novel strains and the reference strains were collected from R2A agar plates that were incubated for 5 days at 30 °C. Fatty acids were extracted and analyzed as reported previously^20^. Fatty acids exceeding 10% of the total fatty acid content were considered the major fatty acids of the strain. Isoprenoid quinones were extracted according to the methods described by Collins and Jones^21^ and were analyzed using high-performance liquid chromatography as reported by Komagata and Suzuki^22^. Polar lipids of the novel strains were extracted according to a previously published method^23^.

### Physiological and morphological characterization

To determine the Gram reaction of strains USB13^T^, AW1^T^, GTP1^T^, and HM2^T^, the non-staining KOH lysis method was employed^24^. Oxidase activity was detected with 1% (w/v) tetramethyl-*p*-phenylenediamine (bioMérieux, Craponne, France) and catalase activity was assessed by observing production of oxygen bubbles after dropping 3% (v/v) hydrogen peroxide (H_2_O_2_) solution. Cell morphology was observed through transmission electron microscopy (TEM) (LIBRA 120; Carl Zeiss) using cells grown in R2A broth for 3 days at 30 °C. Cells were stained with 2% (w/v) uranyl acetate for 2 sec^25^. Growth temperature ranges were assessed by observing colony growth at 4, 7, 10, 15, 20, 25, 28, 30, 35, 37, 40, 42, 45, 50, and 55°C^26^. NaCl tolerance was measured by inoculating strains on R2A agar plates supplemented with various NaCl concentrations (1.0***–***12.0% w/v at 1.0% increments) and incubating them for 7 days at 30 °C. The pH ranges were examined by cultivating strains at 30 °C in R2A broth adjusted to pH 4.0**–**10.0 (at 1.0 pH unit intervals) using citrate/NaH_2_PO_4_ buffer (pH 4.0**–**5.0), phosphate buffer (pH 6.0–8.0), and Tris buffer (pH 9.0**–**10.0), as described in a previous study^27^. Anaerobic growth was assessed by incubating strains for 10 days on R2A plates at 30 °C in a GasPak jar (BBL, Cockeysville, MD, USA). Oxygen absorber strips (Mitsubishi Gas Chemical, Japan) were used to ensure the absence of oxygen. Hydrolysis of carboxymethyl cellulose (CMC; 1%; Duksan), Tween 20 (1%; Biopure), Tween 80 (1%; Biopure), casein (3% skimmed milk; Biopure), starch (1%; Sigma), chitin (1%; Sigma), and DNA (DNase agar; MB cell) was tested as described previously by Smibert and Krieg^28^. Siderophore detection was measured through the blue agar chrome azurol S (CAS) assay^29^. Biochemical characteristics such as enzyme production and substrate assimilation were determined using the API ZYM and API 20NE test systems (bioMérieux).

### Screening of cellulose-degrading strains

To detect their potential cellulolytic activities, the novel strains were inoculated on R2A agar plates supplemented with 1% (w/v) CMC sodium salt and incubated for 7 days at 30 °C. Following incubation, agar plates were stained with 1% (w/v) Congo red dye for 15 min and washed twice with 1 M NaCl solution to visualize the hydrolysis zone^30^. Degradation of CMC was indicated by the formation of clear zones surrounding the colonies. Additionally, for evaluation of their cellulose-degrading ability, strains were inoculated in 100 mL of basal salt medium (K_2_HPO_4_, 2.2 g/L; KH_2_PO_4_, 1.5 g/L; (NH_4_)_2_SO_4_, 1.3 g/L; MgCl_2_, 0.1 g/L; CaCl_2_, 0.02 g/L; FeSO_4_⋅7H_2_O, 0.001 g/L; yeast extract, 5.0 g/L; final pH adjusted to 7.4 with 8 N NaOH)^31^ containing Whatman Grade 1 qualitative filter paper (1 × 6 cm strip, 0.05 g per 20 mL) and incubated in a shaking incubator (160 rpm) at 30 °C for 7 days. All experiments were conducted in triplicate to ensure reproducibility. In addition, only strains showing positive results for both CMC hydrolysis and filter paper degradation were examined in further experiments.

### Phylogenetic and genomic analyses

The 16S rRNA genes of strains USB13^T^, AW1^T^, GTP1^T^, and HM2^T^ were sequenced through Sanger sequencing using the universal bacterial primer sets 27F (5′-AGAGTTTGATCCTGGCTCAG-3′) and 1492R (5′-GGTTACCTTGTTACGACTT-3′) as well as 518F (5′-CCAGCAGCCGCGGTAATAC-3′) and 805R (5′-GACTACCAGGGTATCTAATC-3′), while the sequences were obtained through Sanger sequencing, as previously described by Kim et al.^32^. The nearly complete 16S rRNA genes were assembled using SeqMan software (DNASTAR Inc., Madison, WI, USA), and their similarities to each other, and also to closely related type strains were calculated using National Center for Biotechnology Information (NCBI) Basic Local Alignment Search Tool (BLAST) searches^33^ and the EzBioCloud server (https://www.ezbiocloud.net/)^34^.

Multiple sequences were aligned using the MEGA 7 software^35^, and CLUSTAL_W^36^. Using MEGA 7 software, phylogenetic trees were reconstructed according to the neighbor-joining (NJ)^37^, maximum-likelihood (ML)^38^, and maximum-parsimony (MP)^39^ methods. Tree topology confidence levels were estimated by bootstrap analyses^40^ based on 1,000 replications while evolutionary distances were calculated using Kimura’s two-parameter model^41^. To obtain further taxonomic evidence, a UBCG phylogenomic tree based on the core gene set was reconstructed using the publicly available genomes of closely related taxa^42^. Based on 16S rRNA sequence similarity and phylogenetic analyses, a total of seven type strains of the genus *Ramlibacter* were selected for further chemotaxonomic tests and comparative analyses. *R. monticola* G-3-2^T^, *R. alkalitolerans* CJ661^T^, *R. ginsenosidimutans* BXN5-27^T^, *R. henchirensis* TMB834^T^, and *R. tataouinensis* TTB310^T^ were obtained from the KACC and *R. humi* 18 × 22-1^T^ and *R. rhizophilus* YS3.2.7^T^ were obtained from the KCTC.

Genomic DNA libraries of strains USB13^T^, AW1^T^, GTP1^T^, and HM2^T^ were prepared using the TruSeq Nano DNA Library Prep kit (Illumina, USA) with an insert size of 350 bp. The draft genomes were sequenced by Macrogen Co., Ltd. (Seoul, South Korea) using the HiSeq X platform (Illumina, USA) and assembled using the SOAPdenovo version 3.10.1 de novo assembler^43^. DNA G + C content of the novel strains was calculated from genome data while genome contamination and completeness were assessed using the CheckM bioinformatics tool (https://ecogenomics.github.io/CheckM). ^44^For genome comparison, the known whole genome sequences of the *Ramlibacter* reference strains were obtained from the NCBI database while whole genomes of *R. monticola* KACC 19175^T^, *R. alkalitolerans* KACC 19305^T^, and *R. ginsenosidimutans* KACC 17527^T^ were sequenced and assembled using the same methods as those for the novel strains. Genome similarities of nucleotide sequences among the novel strains and their reference strains were determined by measuring the digital DNA-DNA hybridization (dDDH), average nucleotide identity (ANI), and average amino acid identity (AAI) values. ANI values were calculated using the OrthoANI calculator from EzBioCloud (https://www.ezbiocloud.net/tools/ani) through pairwise comparisons between genome sequences based on the sequence analysis tool, USEARCH^45^, while dDDH values were obtained using the Genome-to-Genome Distance Calculator (GGDC; http://ggdc.dsmz.de/ggdc.php) based on calculations from Formula 2^46^, and AAI values were calculated using the online tool developed by the Kostas lab (http://enve-omics.ce.gatech.edu/aai/index). The draft genome of USB13^T^, AW1^T^, GTP1^T^, and HM2^T^ was annotated using the NCBI Prokaryote Genome Automatic Annotation Pipeline (PGAP), the Rapid Annotation using Subsystems Technology (RAST) server^47^, and the Cluster of Orthologous Groups of proteins (COG) database^48^. Identification of the CAZymes was done using the carbohydrate-active enzymes database (CAZy; http://www.cazy.org/)^49^. The antiSMASH version 5.0 database was used to predict and identify existing secondary metabolism genes and biosynthetic gene clusters within the genome^50^. Additionally, a visual genomic comparison of the genomes of strains USB13^T^, AW1^T^, GTP1^T^, and HM2^T^ was generated using the BLAST Ring Image Generator (BRIG) under default parameters (upper threshold, 70%; lower threshold, 50%; minimum threshold, 50%) with USB13^T^ as the reference genome^51^.

### Microscopy of degraded filter paper

Samples were prepared to visualize morphological interactions between cellulose-degrading bacteria cells and cellulose fiber strands. Strains were first inoculated in 100 mL of basal salt medium containing Whatman Grade 1 qualitative filter paper (1 × 6 cm strip, 0.05 g per 20 mL) and incubated for 14 days at 30 °C. Following incubation, the degraded filter paper and bacterial cell mixture was loaded on round glass cover slips (Marienfeld, Germany) and fixed with 2.5% glutaraldehyde in 1X phosphate buffered saline (PBS; NaCl, 8 g/L; KCl, 0.2 g/L; Na_2_HPO_4_, 1.44 g/L; KH_2_PO_4_, 0.24 g/L; pH 7.4), according to the methods described by Ji et al.^52^. Subsequently, samples were washed three times with PBS buffer, followed by dehydration using a graded series of ethanol (30–100%). Samples were coated with platinum (15 nm; EM ACE200, Leica, Wetzlar, Germany) and examined under field emission-scanning electron microscopy (FE-SEM) analysis.

### Crude enzyme production and enzymatic assay

Strains that showed positive results for CMC hydrolysis were cultured in basal salt medium containing Whatman Grade 1 qualitative filter paper (1 × 6 cm strip, 0.05 g per 20 mL) as the carbon source. Strains were incubated for a total of 7 days where cellulase activity was to be measured at 1, 3, 5, and 7 days following initial inoculation. On the days of testing, supernatant was collected and centrifuged at 5000 rpm for 15 min at 4 °C for enzyme activity analysis.

The activities of endoglucanase, exoglucanase, *β*-glucosidase, and total cellulase were determined by examining the amount of reducing sugar (glucose) released from CMC sodium salt, avicel, salicin, and filter paper, respectively. Enzyme activities were assessed in buffer solutions with different pH values (6.0, 7.0, 8.0) according to the dinitrosalicylic acid (DNS) method described by Ghose^53^. Endoglucanase, exoglucanase, and *β*-glucosidase activities were measured by incubating a mixture of 0.05 M citrate buffer, 0.5 mL of crude enzyme supernatant, and the corresponding substrates for 30 min at 50 °C. Total cellulase activity was measured by incubating 0.5 mL of crude enzyme supernatant with 1.0 mL of 0.05 M citrate buffer and one strip of Whatman Grade 1 qualitative filter paper (1 × 6 cm, approximately 50 mg) for 60 min at 50 °C. After incubation, the substrate hydrolysis reaction was terminated by adding 3 mL of DNS reagent. Results were measured spectrophotometrically where glucose standards were used to approximate the amount of glucose produced. All spectrophotometric data were measured using a UV–Vis spectrophotometer (U-3310, Hitachi, Tokyo, Japan). Enzyme activity was defined in international units (IU or U); one unit of enzymatic activity was defined as the amount of enzyme that releases 1 μmol of glucose per mL per 1 min of reaction. All experiments were conducted in triplicate and the data obtained were presented as mean values ± standard deviation.

## Results and discussion

### Chemotaxonomic characteristics

The predominant respiratory quinone for all novel strains was ubiquinone 8 (Q-8), consistent with other *Ramlibacter* species. C_16:0_ and summed feature 3 (consisting of C_16:1_ ω7c and/or C_16:1_ ω6c) were identified as the common major fatty acids (> 10%) of the novel strains USB13^T^, AW1^T^, GTP1^T^, and HM2^T^. Other than the aforementioned fatty acids, strain USB13^T^ had C_10:0_ 3-OH additionally as its major fatty acid, whereas strains AW1^T^ and HM2^T^ shared C_17:0_ cyclo and summed feature 8 (consisting of C_18:1_ ω7c and/or C_18: 1_ ω6c) as its additional fatty acids. Detailed comparisons of the fatty acid profiles of the novel strains and their reference strains are summarized in Table S1.

Strains USB13^T^, AW1^T^, GTP1^T^, and HM2^T^ shared major polar lipids diphosphatidylglycerol (DPG), phosphatidylglycerol (PG), and phosphatidylethanolamine (PE), which was consistent with the major polar lipids of the reference strains. Additionally, the polar lipid profile of USB13^T^ consisted of one unidentified phosphoaminolipid, two unidentified phosphoglycoaminolipids, and six unidentified polar lipids while the polar lipid profile of AW1^T^ had one unidentified lipid, one unidentified phosphoglycolipid, and three unidentified glycolipids in addition. The polar lipid profile of strain GTP1^T^ additionally consisted of two unidentified phosphoaminolipids, and that of strain HM2^T^ additionally had one unidentified phosphoaminolipid, one unidentified phosphoglycolipid, one unidentified phosphoglycoaminolipid, and two unidentified phospholipids. Polar lipid profiles of the novel strains USB13^T^, AW1^T^, GTP1^T^, and HM2^T^ are shown in Figure S1.

### Physiological, morphological characteristics, and screening of cellulose-degrading strains

When grown on R2A agar, strain USB13^T^ produced reddish white and flat colonies while strain AW1^T^ produced orange, convex colonies, strain GTP1^T^ produced white, convex colonies, and strain HM2^T^ produced cream-colored, flat, transparent colonies. Under TEM, monotrichous flagella were observed only in strain HM2^T^, and when tested for motility, strain USB13^T^ and AW1^T^ showed gliding motility, whereas strain GTP1^T^ was non-motile. Strains USB13^T^ and HM2^T^ showed positive results for both catalase and oxidase activities; strain AW1^T^ showed positive results for catalase and negative results for oxidase activity, and strain GTP1^T^ showed negative results for catalase and positive results for oxidase activity. All strains were identified to be strictly aerobic, while showing negative results for urea, gelatin, starch, chitin, and DNA hydrolysis and positive results for hydrolysis of Tween 80. In addition, strain USB13^T^ was the only strain to produce iron-chelating siderophores. When tested for NaCl tolerance, growth of strain USB13^T^ was observed in NaCl concentrations of 0–7% (w/v), possibly due to the fact the strain was isolated from a marine environment. A detailed comparison of physiological and morphological characteristics between the novel species and its closely related *Ramlibacter* strains is presented in Table 1, while TEM images of the novel strains are shown in Figure S2. Results of the reference strains in Table 1 coincided with the data from the original literature^1,3–5,7,8^.

**Table 1:** Characteristics differentiating *strains* USB13^T^, AW1^T^, GTP1^T^, and HM2^T^ from closely related strains of the genus *Ramlibacter*.

Characteristics	1	2	3	4	5	6	7	8	9	10	11
Motility*	Non-motile	Non-motile	Non-motile	Monotri-chous Flagella	Non-motile	Non-motile	Peritri-chous Flagella	Non-motile	Gliding Motility	Gliding Motility	Non-motile
Temperature range (°C)	7–50	7–45	10–45	7–45	15–37	25–37	25–37	15–37	25–40	25–40	10–40
NaCl tolerance (%)	0–7	0–3	0–2	0–3	0–0.5	0	0–1	0–1	0–2	0–2	0–5
Oxidase/catalase activity	+/+	−/+	+/−	+/+	−/+	+/+	+/+	+/+	+/+	+/+	+/+
**Hydrolysis of:**											
Casein	−	−	−	+	−	+	−	−	−	−	−
CM-cellulose	+	−	−	−	−	−	−	−	−	w+	+
DNA	−	−	−	−	−	−	−	−	−	−	+
Esculin	+	+	−	w+	w+	+	−	−	+	+	−
Gelatin	−	−	−	−	−	+	−	−	+	−	−
Starch	−	−	−	−	−	−	−	+	−	−	+
Tween 20	+	−	+	−	+	+	+	−	−	+	−
Tween 80	+	+	+	+	−	+	−	+	+	+	−
Urea	−	−	−	−	+	−	−	−	−	−	+
**Enzyme activities (API ZYM):**											
Acid phosphatase	+	−	−	+	+	−	−	+	+	−	+
Alkaline phosphatase	+	+	+	+	+	−	−	+	+	−	+
*α*-Chymotrypsin	−	−	−	−	−	−	−	−	+	+	+
Cystine arylamidase	−	−	−	−	−	+	−	w+	+	+	+
Esterase (C4)	−	w+	+	+	−	+	+	+	+	+	+
*α*-Galactosidase	−	−	−	−	−	+	−	−	−	−	−
*β*-Galactosidase	+	−	−	−	−	−	+	−	+	−	−
*α*-Glucosidase	+	−	−	+	−	−	−	−	−	−	−
*β*-Glucosidase	+	+	−	−	−	−	+	−	+	+	−
Leucine arylamidase	+	+	+	+	+	−	−	+	+	w+	+
Lipase (C14)	−	−	−	−	−	+	−	−	−	−	+
Naphthol-AS-BI-phosphohydrolase	−	−	−	w+	w+	+	−	+	+	+	+
Trypsin	−	−	−	−	−	−	−	−	+	+	+
Valine arylamidase	−	−	−	+	w+	+	−	w+	+	+	+
**Assimilation of (20 NE):**											
D-Glucose	+	−	−	−	−	+	+	−	+	−	−
D-Mannitol	−	−	−	−	−	+	−	−	−	−	−
Maltose	−	−	−	−	−	+	−	−	−	−	−
DNA G + C content (%) †	69.7	68.6	67.9	69.9	69.3	69.2	68.7	68.9	68.5	70.0	69.7

* Data from original literature^1,3–5,7,8^.

^†^From NCBI Genome Database (https://www.ncbi.nlm.nih.gov/genome).

Strains: 1, USB13^T^; 2, AW1^T^; 3, GTP1^T^; 4, HM2^T^; *R. monticola* KACC 19175^T^; 6, *R. alkalitolerans* KACC 19305^T^; 7, *R. ginsenosidimutans* KACC 17527^T^; 8, *R. humi* KCTC 52922^T^; 9, *R. henchirensis* KACC 11925^T^; 10, *R. tataouinensis* KACC 11924^T^; 11, *R. rhizophilus* KCTC 52083^T^. All strains are positive for esterase lipase (C8), while all strains are negative for chitin hydrolysis. All data were obtained from this study unless indicated otherwise. + , Positive; w + , weakly positive; -, negative.

R2A agar plates supplemented with 1% (w/v) CMC were stained with Congo red dye after 7 days of incubation. Clear zones only formed around colonies of strain USB13^T^, indicating that strain USB13^T^ solely possessed CMC-hydrolyzing activity among the four novel strains. When inoculated in basal salt medium, filter paper from the USB13^T^ sample underwent degradation, whereas samples containing strains AW1^T^, GTP1^T^, and HM2^T^ did not show any signs of degradation.

### Phylogenetic and genomic analyses

**Figure 1:**
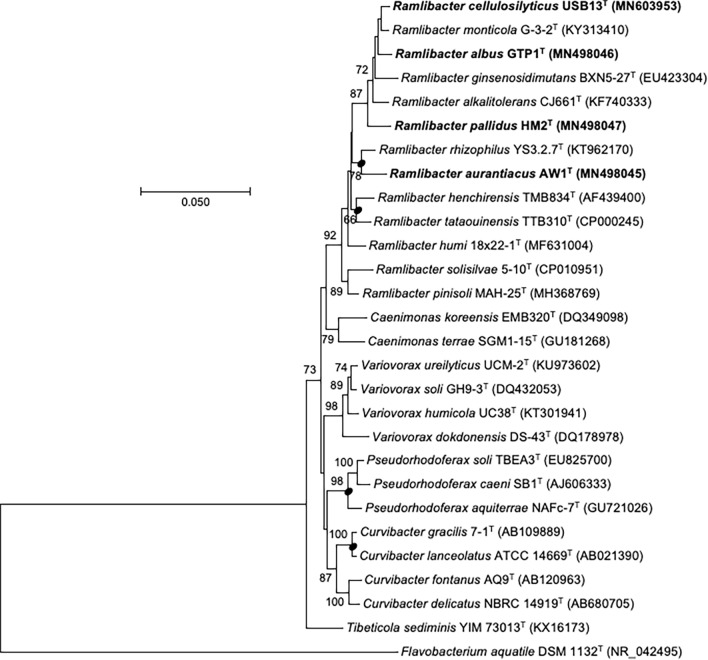
Maximum-parsimony (MP) tree reconstructed based on 16S rRNA gene sequences, showing the relationship between strains USB13^T^, AW1^T^, GTP1^T^, and HM2^T^ and other closely related type strains. Bootstrap values based on 1000 replications are listed as percentages at branching points. Only bootstrap values exceeding 50% are shown. Bar, 50 substitutions per nucleotide position.

**Figure 2:**
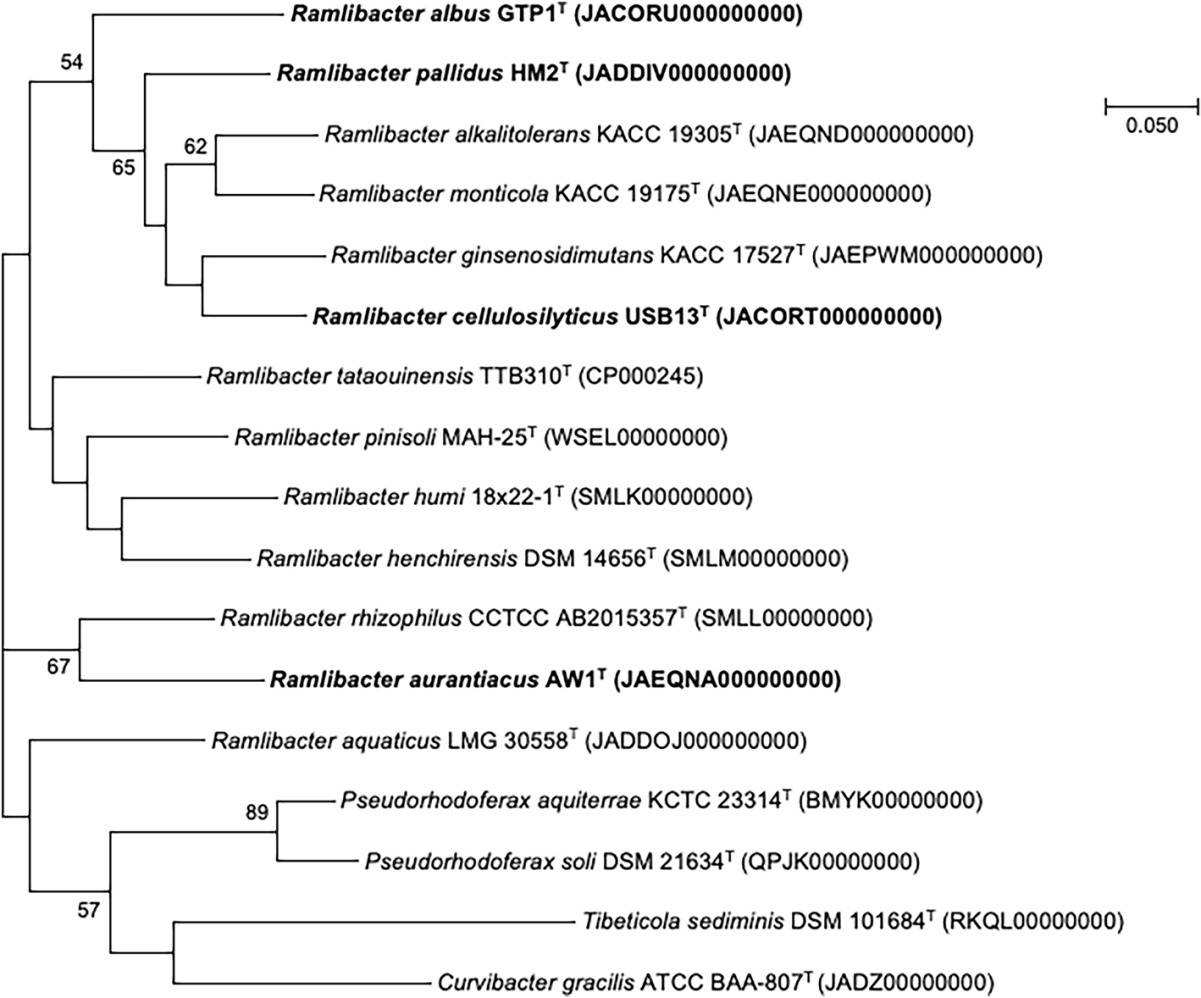
Phylogenomic tree of strains USB13^T^, AW1^T^, GTP1^T^, and HM2^T^ and their closely related taxa was reconstructed based on core genomes using UBCG version 3.0 pipeline^42^. NCBI GenBank accession numbers are shown in parentheses. Bootstrap analysis was carried out using 1000 replications. Percentage bootstrap values (> 50%) are given at branching points. Bar, 0.050 substitution per position.

EzBioCloud search results and BLASTn searches revealed that the novel strains belonged to the family *Comamonadaceae* and genus *Ramlibacter*. Using BLASTn, 16S rRNA gene sequence similarities were determined where strain USB13^T^ was closest to strain GTP1^T^ (98.5%), followed by strain HM2^T^ (98.1%) and strain AW1^T^ (97.1%). Strain AW1^T^ shared the highest similarity with strain GTP1^T^ (97.3%), followed by strain HM2^T^ (97.1%), while strain GTP1^T^ shared a similarity of 98.2% with strain HM2^T^. Phylogenetic analysis based on the MP method (Fig. 1) showed the clustering of the novel strains USB13^T^, AW1^T^, GTP1^T^, and HM2^T^ with strains such as *R. monticola* G-3-2^T^, *R. ginsenosidimutans* BXN5-27^T^, *R. alkalitolerans* CJ661^T^, and *R. rhizophilus* YS3.2.7^T^. Similar topologies were observed in trees reconstructed by ML (Figure S3) and MP methods. The UBCG phylogenomic tree (Fig. 2), which was reconstructed using whole genome sequences, also showed close clustering of the selected reference strains and novel strains.

**Figure 3:**
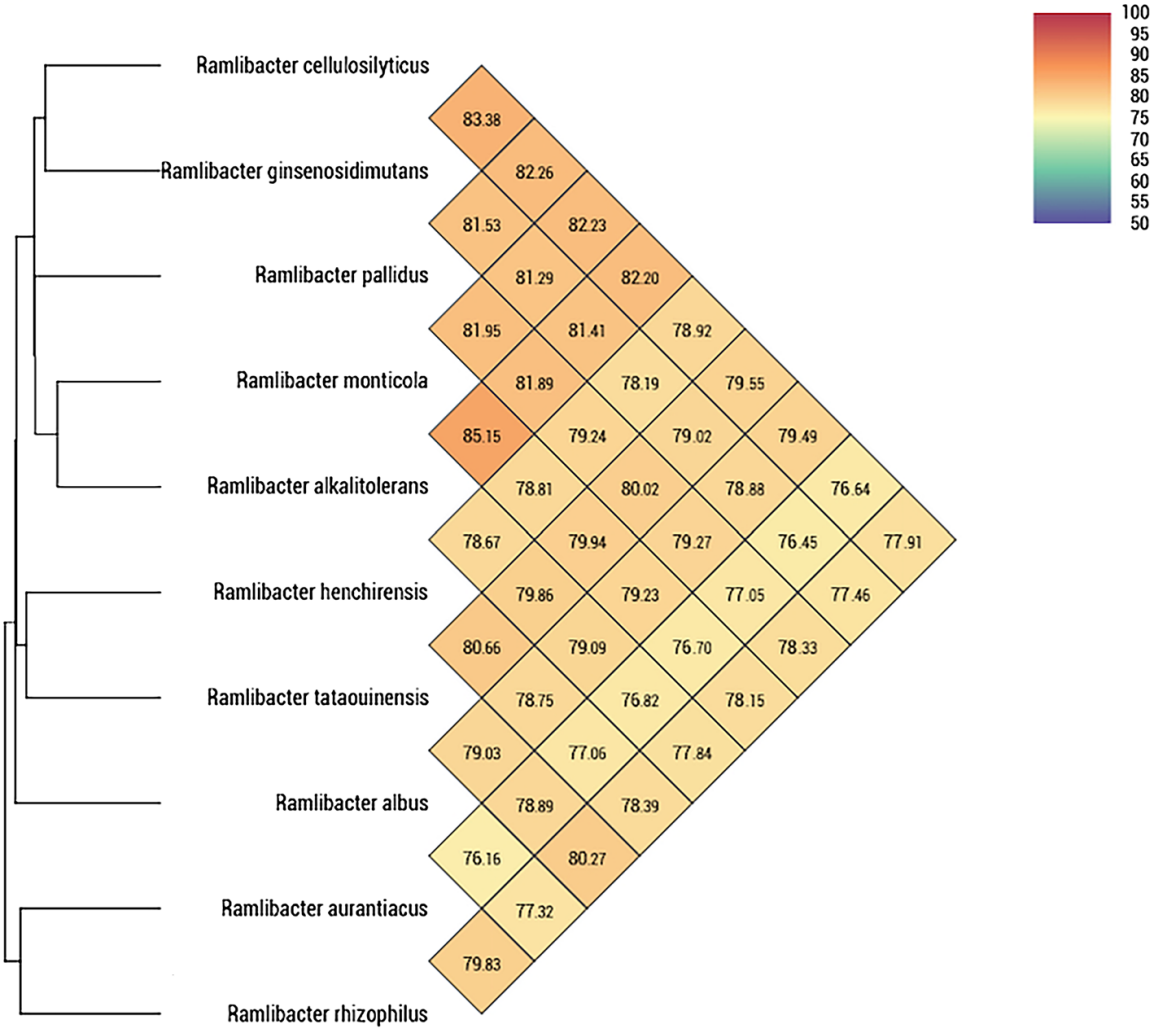
Heatmap of strains USB13^T^, AW1^T^, GTP1^T^, and HM2^T^ and other closely related strains within the genus *Ramlibacter*, generated with OrthoANI values calculated using OAT software^45^. Bacterial strains and accession numbers are indentical to those of Fig. 2.

Draft genome sequences of the novel strains USB13^T^, AW1^T^, GTP1^T^, and HM2^T^ were deposited in the GenBank database under the accession numbers JACORT000000000, JAEQNA000000000, JACORU000000000, and JADDIV000000000, respectively. In addition, the draft genome sequences of *R. monticola* KACC 19175^T^, *R. alkalitolerans* KACC 19305^T^, and *R. ginsenosidimutans* KACC 17527^T^ were also deposited in GenBenk under the accession numbers JAEQNE000000000, JAEQND000000000, and JAEPWM000000000, respectively. The assembled genome size of the novel strains USB13^T^, AW1^T^, GTP1^T^, and HM2^T^ was 5.53 Mbp, 5.11 Mbp, 6.15 Mbp, 4.31 Mbp, respectively. G + C content ranged from 67.9% to 69.9%, which was similar to those of the reference strains. The genomic features of the novel strains and their closely related *Ramlibacter* strains are presented in Table S2. CheckM analysis showed the following estimations for each strain: USB13^T^, had a 99.84% completeness and 0.68% contamination; AWI^T^, had a 99.84% completeness and 0.86% contamination; GTP1^T^, had a 99.38% completeness and 1.32% contamination; HM2^T^, had a 97.51% completeness and 0.16% contamination. These results indicated that the draft genome results for all strains were reliable. ANI values between the novel strains and reference strains ranged from 76.5–83.4% while dDDH values ranged from 20.7–26.7%, and AAI values ranged from 65.7–80.4%. All values were below the threshold for delineation of a new species^54^. ANI values between the novel strains and their reference strains are presented in Fig. 3, while a detailed comparison of GGDC and AAI values are shown in Table 2.

**Table 2:** Average amino acid identity (AAI) and digital DNA-DNA hybridization (dDDH) value comparisons between the closely related *Ramlibacter* type species and the novel strains, USB13^T^, AW1^T^, GTP1^T^, and HM2^T^. AAI values were calculated by two-way AAI, while dDDH values were calculated based on formula 2^46^.

Reference Strains	dDDH values (%)				AAI values (%)			
	USB13^T^	AW1^T^	GTP1^T^	HM2^T^	USB13^T^	AW1^T^	GTP1^T^	HM2^T^
USB13^T^	–	21.1	23.0	25.3	–	67.3	71.9	78.9
AW1^T^	–	–	20.7	21.1	–	–	65.7	68.1
GTP1^T^	–	–	–	22.3	–	–	–	72.0
*R. monticola* KACC 19175^T^	25.6	21.2	22.6	25.1	76.9	65.9	70.8	76.4
*R. alkalitolerans* KACC 19305^T^	25.1	21.1	22.5	25.0	77.3	66.2	70.9	76.4
*R. ginsenosidimutans* KACC 17527^T^	26.7	21.0	22.4	24.7	80.4	66.8	70.5	76.8
*R. humi* 18 × 22-1^T^	22.4	21.2	22.0	22.3	71.9	68.3	71.3	71.9
*R. henchirensis* DSM 14656^T^	22.1	21.1	21.9	22.0	72.0	68.6	70.7	72.4
*R. tataouinensis* TTB310^T^	22.6	22.5	22.1	22.8	71.9	71.0	70.9	73.0
*R. rhizophilus* CCTCC AB2015357^T^	21.6	23.3	21.3	21.4	69.5	74.2	67.2	69.9

Based on NCBI PGAP annotation and CAZyme prediction results, strain USB13^T^, which was the only strain to show cellulolytic activity, possessed a total of four protein CDs encoding CAZymes, namely, two GH15 proteins, one glycosyl hydrolase protein, and one GH99-like domain-containing protein. Despite not showing any cellulolytic activity, strain AW1^T^ possessed eight CAZyme CDs; the most amount among the novel strains. The enzymes include, two GH2 proteins, one GH5 protein, three GH15 proteins, one glycoside hydrolase protein, and one cellulase family glycosyl hydrolase. Strain GTP1^T^ possessed two CDs encoding one GH15 protein and one GH16 protein; strain HM2^T^ possessed three CDs encoding one GH2, one GH15, and one GH18 protein. All strains possessed GH15, which is known for its glucoamylase activity in fungi^55^. A detailed summary of the novel strains CAZymes are presented in Table S3 and a comparison of CAZyme numbers between strains USB13^T^, AW1^T^, GTP1^T^, and HM2^T^ is summarized in Table S4. The presence of these genes may suggest the cellulolytic activity of strain USB13^T^, while it is uncertain why GH families responsible for endoglucanase (GH 5–8, 12, 16, 44, 45, 48, 51, 64, 71, 74, 81, 87, 124, and 128), exoglucanase (GH 5–7, and 48), and *β*-glucosidase (GH 1, 3, 4, 17, 30, and 116) were not present in the genome^11^.

**Figure 4:**
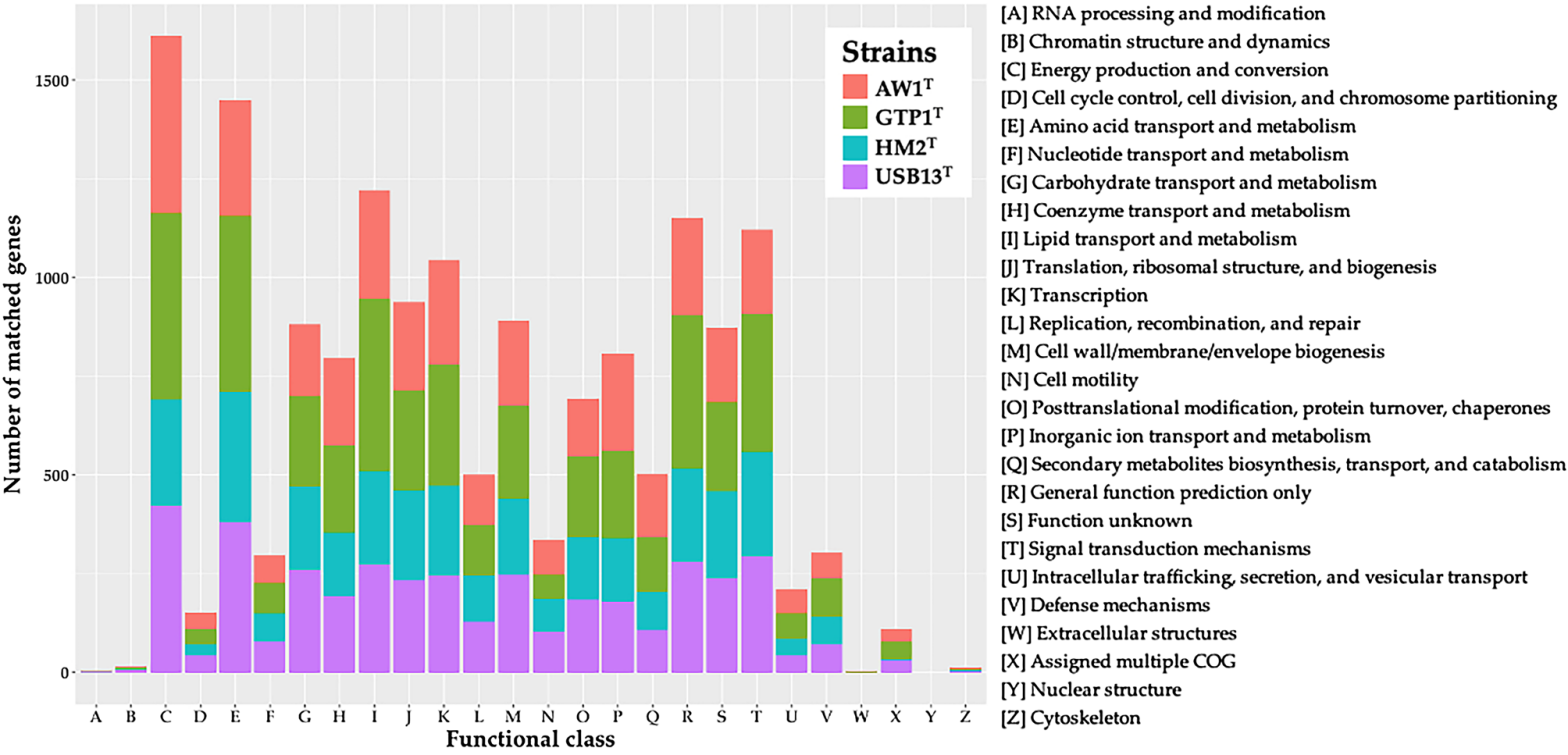
Comparison of total number of matched genes of strains USB13^T^, AW1^T^, GTP1^T^, and HM2^T^ according to functional classes based on Cluster of Orthologous Groups of proteins (COG) predictions^48^.

COG predictions (Fig. 4) revealed that the majority of the core genes of the four novel strains accounted for genes belonging to the functional categories C (energy production and conversion), E (amino acid transport and metabolism), I (lipid transport and metabolism), T (signal transduction mechanisms), and K (transcription). Meanwhile, the number of core genes belonging in category G, carbohydrate transport and metabolism, was the highest for strain USB13^T^ (258), followed by GTP1^T^ (230), HM2^T^ (212), and AW1^T^ (181). The high number of genes in strain USB13^T^ may be a contributing factor in the strain’s cellulolytic activity. A comparison of COG gene count distribution of the novel strains is presented in Table S5.

AntiSMASH analysis results showed four gene clusters within the genome of strain USB13^T^: ribosomally synthesized and post-translationally modified peptides (RIPP)-like cluster (989,516–1,000,916 nt; JACORT010000001), terpene synthesis (8,622–30,347 nt; JACORT010000003), RIPP precursor peptide recognition element (RRE)-containing cluster (311,469–333,619 nt; JACORT010000004), and redox-cofactor (281,860–303,948 nt; JACORT010000007). Among the clusters, the RRE-containing cluster showed 11% similarity to streptobactin, a tricatechol-type siderophore isolated from Streptomyces sp. YM5-799^56^. Strain AW1^T^ had a total of eight gene clusters which encoded for: arylpolyene (165,946–207,130 nt), terpene (618,322–640,854 nt), RIPP-like proteins (804,411–819,137 nt), non-ribosomal peptide synthetase cluster (NRPS)-like (61,798–104,764 nt), betalactone (323,399–348,739 nt), N-acetylglutaminylglutamine amide (NAGGN; 106,834–121,648 nt), type I polyketide synthase (T1PKS; 56,584–107,578 nt), and heterocyst glycolipid synthase-like polyketide synthase (hglE-KS; 75,419–113,566 nt). Strain GTP1^T^ possessed four gene clusters that encoded for RRE-containing cluster (175,155–199,102 nt), homoserine lactone (110,293–130,892 nt), a signaling molecule known for its involvement in bacterial quorum sensing, the RIPP-like cluster (38,002–48,856 nt), and terpene synthesis (47,942–69,701 nt). Strain HM2^T^ had two gene clusters that encoded for resorcinol (403,967–445,901 nt), an organic compound known for its antiseptic properties, and terpene (697,660–721,242 nt), which showed 100% similarity for carotenoid synthesis. BRIG analysis results showed that a majority of the regions within the four analyzed genomes were conserved with at least 70% similarity (Figure S4).

### Cellulolytic potential and FE-SEM analysis of strain USB13^T^

**Figure 5:**
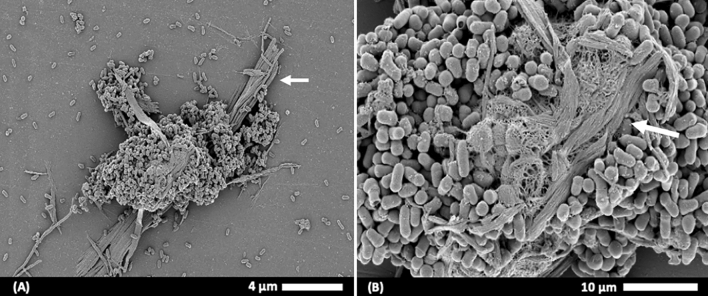
Field emission-scanning electron microscopy (FE-SEM) images of adhesion of strain USB13^T^ to degraded filter paper fibers. Arrows indicate filter paper fibers. (**A**) low magnification (5000$\times$) and (**B**), high magnification (20,000$\times$) images of strain USB13^T^ surrounding filter paper fibers.

A USB13^T^-inoculated basal salt medium sample containing degraded filter paper was examined under FE-SEM to observe the morphological interactions between cellulose fibers and USB13^T^ cells. Images in Fig. 5 show individual rod cells of strain USB13^T^ surrounding filter paper fibers, indicating bacterial adherence.

**Figure 6:**
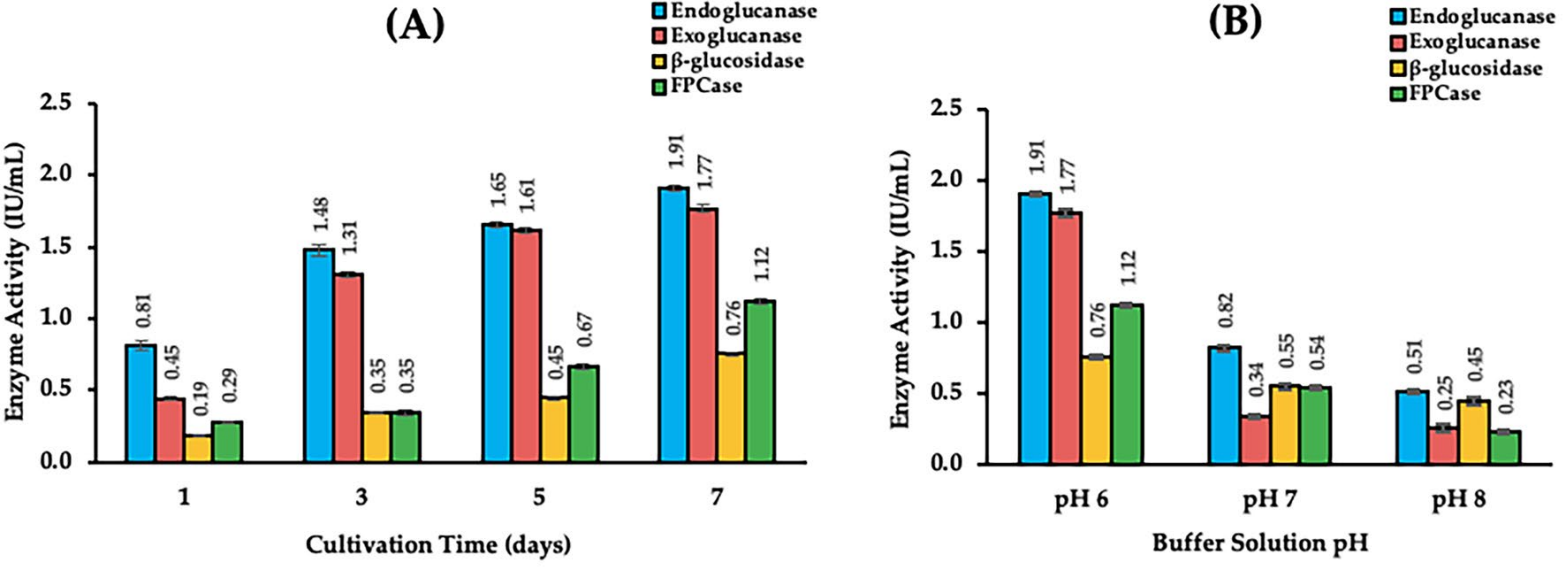
Cellulolytic enzyme activity of strain USB13^T^. Enzyme activity was defined in international units (IU); one unit of enzymatic activity was defined as the amount of enzyme that releases 1 μmol of glucose per mL per 1 min of reaction. (**A**) cellulase activity results under different cultivation time; (**B**) cellulase activity under different buffer solution pH. Values in the figure are mean values of triplicate data with standard deviation.

The enzymatic assay results showed endoglucanase, exoglucanase, *β*-glucosidase, and filter paper cellulase (FPCase) activities of strain USB13^T^, wherein activities for endoglucanase was the highest and *β*-glucosidase was the lowest in all experiments. As seen in Fig. 6A, enzyme activity for all cellulolytic enzymes increased along with its cultivation time. In addition, enzyme activities showed the highest results when tested on buffer solutions of pH 6.0 (Fig. 6B), indicating the enzymes’ resistance to moderately acidic conditions. The pH of the buffer solution seemed to be an important factor in enzyme activity, as activity of endoglucanase, exoglucanase, and FPCase drastically decreased when the pH was altered from pH 6.0 to pH 7.0. Meanwhile, *β*-glucosidase activity was relatively resistant to pH change as its activity decreased less than 50%. On day 7, enzyme activities were measured as 1.91 IU/mL for endoglucanase, 1.77 IU/mL for exoglucanase, 0.76 IU/mL for *β*-glucosidase, and 1.12 IU/mL for FPCase at pH 6.0. When measured at pH 8.0, where enzyme activity was the lowest, enzyme activities were measured as 0.51 IU/mL for endoglucanase, 0.25 IU/mL for exoglucanase, 0.45 IU/mL for *β*-glucosidase, and 0.23 IU/mL for FPCase; all values were less than half of the measured activity at pH 6.0. The results of strain USB13^T^ are comparable to FPCase results of other species such as *Mucilaginibacter polytrichastri* RG4-7^T^ (0.98 U/mL) isolated from the moss *Polytrichastrum formosum*^14^, *Paenibacillus lautus* BHU3 (2.9 U/mL) isolated from a landfill site^57^, and *Serratia rubidaea* DBT4 (0.5 U/mL) isolated from the gastrointestinal tract of a black Bengal goat^58^.

Despite the absence of the main three cellulolytic enzymes, endoglucanase, exoglucanase, and *β*-glucosidase, the cellulolytic activity of strain USB13^T^ was confirmed through SEM images, CMC agar screening, and enzymatic assay results. However, because PGAP annotation results showed that other non-cellulolytic strains also possessed CAZymes, in some cases more than strain USB13^T^, further research is necessary to understand the mechanics of how CAZymes and other cellulases interact to degrade cellulose, and how these genes are expressed under certain conditions. Furthermore, the cellulolytic activity of strain USB13^T^ can be further optimized for commercial use by adjusting growth conditions such as pH, temperature, and growth media.

While cellulolytic bacteria are known to inhabit animal intestinal tracts, the rumen, and soil, they can be found almost everywhere, such as ocean floors, municipal landfills, and even extreme environments such as hot springs^59^. In these habitats, cellulolytic bacteria utilize cellulose while cohabiting with non-cellulolytic bacteria. There have been many studies suggesting the synergistic role non-cellulolytic bacteria play in cellulose degradation, where non-cellulolytic bacteria aid cellulose degradation by neutralizing pH or removing harmful metabolites^60–62^.

Bacterial cellulases have shown immense value in various industries such as animal feed processing, food and brewery production, and agriculture, not to mention biofuel synthesis through biomass utilization^11^. Due to the versatile uses of bacterial cellulases, the cellulolytic strain USB13^T^ has the potential to become an invaluable resource. However, further research of the novel strain’s cellulose-degradation mechanisms is necessary to develop and commercially make use of its bacterial cellulases in the future. In addition, research regarding co-culturing non-cellulolytic bacteria and strain USB13^T^ may also help in developing effective methods to use an otherwise underutilized bioresource.

### Taxonomy of novel *Ramlibacter* species

While phylogenetic analyses indicated that the novel strains USB13^T^, AW1^T^, GTP1^T^, and HM2^T^ should be assigned to the genus *Ramlibacter*, differences in fatty acid compositions, polar lipid profiles, and physiological characteristics suggested that the four novel strains are noticeably distinct from other validly published species of the genus. Additionally, genomic characteristics such as ANI, dDDH, and AAI values further supported the novel strains’ position as a distinct species within the genus *Ramlibacter*. Therefore, we propose that the strains USB13^T^, AW1^T^, GTP1^T^, and HM2^T^ represent novel species within the genus *Ramlibacter*.

### Description of the novel* Ramlibacter* species

The descriptions of the novel species are given according to the standards of the Judicial Commission of the International Committee on Systematic Bacteriology^63^.

### Description of *Ramlibacter cellulosilyticus sp. nov*

*Ramlibacter cellulosilyticus* (cel.lu.lo.si.ly'ti.cus. N.L. n. *cellulosum*, cellulose; N.L. adj. *lyticus* from Gr. *lytikos*, dissolving; N.L. masc. adj. *cellulosilyticus*, cellulose-dissolving).

Cells of strain USB13^T^ are Gram-negative, rod-shaped, non-flagellated and motile by gliding. The strain is positive for both oxidase and catalase activity, while cells have a width of 0.3–0.5 μm and length of 2.0–2.4 μm. When observed on R2A agar, colonies are reddish white, flat with entire margins, and have a diameter of 1–2 mm. Growth of strain USB13^T^ is observed at 7–50 °C (optimum, 28–30 °C), at pH 5.0–10.0 (optimum, pH 6.0), and at NaCl concentrations of 0–7% (optimum, 0–3%). The strain is unable to grow in anaerobic conditions. Produces siderophores and hydrolyzes Tween 20, Tween 80, CMC, and esculin. According to the API ZYM results, the strain showed positive results for alkaline phosphatase, esterase lipase (C8), leucine arylamidase, acid phosphatase, *β*-galactosidase, *α*-glucosidase, and *β*-glucosidase. In the API 20NE assay, strain USB13^T^ showed positive results only for *β*-galactosidase. The predominant respiratory quinone is ubiquinone 8 (Q-8). The major fatty acids are C_16:0_, C_10:0_ 3-OH, and summed feature 3 (consisting of C_16:1_ ω7c and/or C_16:1_ ω6c). The polar lipid profile consists of diphosphatidylglycerol (DPG), phosphatidylglycerol (PG), phosphatidylethanolamine (PE), one unidentified phosphoaminolipid, two unidentified phosphoglycoaminolipids, and six unidentified polar lipids. The G + C content is 69.7%. The GenBank/EMBL/DDBJ accession numbers for the 16S rRNA gene sequence and the assembled genome sequence of strain USB13^T^ are MN603953 and JACORT000000000, respectively.

The type strain USB13^T^ (= KACC 21656^T^ = NBRC 114839^T^) was isolated from shallow coastal water at Haeundae Beach, Busan, Republic of Korea.

### Description of *Ramlibacter aurantiacus sp. nov*

*Ramlibacter aurantiacus* (au.ran.ti'a.cus. L. masc. adj. *aurantiacus*, orange-colored, referring to the orange colonies of the strain).

Cells of strain AW1^T^ are Gram-negative, coccoid to short rod-shaped, non-flagellated, and motile by gliding. The strain is negative for oxidase activity, and positive for catalase activity. When observed on R2A agar, colonies are orange, convex, with entire margins, and 0.5–1.0 mm in diameter. Under TEM cells have and approximate width of 0.3–0.5 μm and length of 0.6–0.8 μm. Growth of strain AW1^T^ can be observed at 7–45 °C (optimum, 30 °C), at pH 7.0–10.0 (optimum, 7.0–8.0), and at NaCl concentrations of 0–3% (optimum, 0–1%). The strain does not grow under anaerobic conditions but is able to hydrolyze Tween 80. In addition, AW1^T^ is not able to produce siderophores. In the API ZYM assay, positive for alkaline phosphatase, esterase (C4), esterase lipase (C8), leucine arylamidase, and *β*-glucosidase. In the API 20NE assay, positive for esculin hydrolysis. The predominant respiratory quinone is ubiquinone 8 (Q-8). The major fatty acids are C_16:0_, C_17:0_ cyclo, summed feature 3 (consisting of C_16:1_ ω7c and/or C_16:1_ ω6c), and summed feature 8 (consisting of C_18:1_ ω7c and/or C_18:1_ ω6c). The polar lipid profile consists of diphosphatidylglycerol (DPG), phosphatidylglycerol (PG), phosphatidylethanolamine (PE), one unidentified phosphoglycolipid, one unidentified lipid, and three unidentified glycolipids. The G + C content is 68.6%. The GenBank/EMBL/DDBJ accession numbers for the 16S rRNA gene sequence and the assembled genome sequence of strain AW1^T^ are MN498045 and JAEQNA000000000, respectively.

The type strain AW1^T^ (= KACC 21544^T^ = NBRC 114862^T^) was isolated from soil at Aewol, Jeju Island, Republic of Korea.

### Description of *Ramlibacter albus sp. nov*

*Ramlibacter albus* (al'bus. L. masc. adj. *albus*, white, referring to the white colonies of the strain).

Strain GTP1^T^ is non-motile, Gram-negative, strictly aerobic, positive for oxidase activity, and negative for catalase activity. When observed on R2A, colonies are white, convex, with entire margins, and 1–2 mm in diameter. Under TEM, cells lack flagella, are rod-shaped, and have a width of 0.7–0.8 μm and length of 1.6–1.9 μm. Growth of strain GTP1^T^ can be observed at 10–45 °C (optimum, 30 °C), at pH 5.0–8.0 (optimum, pH 7.0), and at NaCl concentrations of 0–2% (optimum, 0%). The strain shows positive results for Tween 20 and Tween 80 hydrolysis. GTP1^T^ does not produce siderophores when tested on CAS-blue agar. According to API ZYM results, strain GTP1^T^ is positive for alkaline phosphatase, esterase (C4), esterase lipase (C8), and leucine arylamidase, while the API 20NE assay results show negative results for all substrates. The predominant respiratory quinone is ubiquinone 8 (Q-8). The major fatty acids are C_16:0_ and summed feature 3 (consisting of C_16:1_ ω7c and/or C_16:1_ ω6c). The polar lipid profile consists of diphosphatidylglycerol (DPG), phosphatidylglycerol (PG), phosphatidylethanolamine (PE), and two unidentified phosphoaminolipids. The predominant respiratory quinone is ubiquinone 8 (Q-8). The major fatty acids are C_16:0_, C_17:0_ cyclo, summed feature 3 (consisting of C_16:1_ ω7c and/or C_16:1_ ω6c), and summed feature 8 (consisting of C_18:1_ ω7c and/or C_18:1_ ω6c). The polar lipid profile consists of diphosphatidylglycerol (DPG), phosphatidylglycerol (PG), phosphatidylethanolamine (PE), one unidentified phosphoaminolipid, one unidentified phosphoglycolipid, one unidentified phosphoglycoaminolipid, and two unidentified polar lipids. The G + C content is 67.9%. The GenBank/EMBL/DDBJ accession numbers for the 16S rRNA gene sequence and the assembled genome sequence of strain GTP1^T^ are MN498046 and JACORU000000000, respectively.

The type strain GTP1^T^ (= KACC 21702^T^ = NBRC 114488^T^) was isolated from soil at Seogwipo, Jeju Island, Republic of Korea.

### Description of *Ramlibacter pallidus sp. nov*

*Ramlibacter pallidus* (pal'li.dus. L. masc. adj. *pallidus*, pale, referring to the color of the colonies).

Cells of strain HM2^T^ are Gram-negative, and positive for both oxidase and catalase activities. When observed on R2A agar, colonies are cream-colored, transparent, 1.0–2.5 mm in diameter, and flat with entire margins. Under TEM, monotrichous flagella are observed, and cells are rod-shaped with a width of 0.4–0.78 μm and length of 1.7–1.8 μm. The strain shows the fastest growth at a temperature range of 25–35 °C and at pH values between 8.0 and 9.0. When NaCl is present, growth is observed at concentrations of 0–3% (w/v), with optimal growth was observed at concentrations of 0–1% (w/v). The strain is not able to tolerate anaerobic conditions. Strain HM2^T^ hydrolyzes Tween 80 and weakly hydrolyzes casein. However, siderophore production cannot be observed when tested on CAS-blue agar. According to API ZYM tests, strain HM2^T^ shows positive results for alkaline phosphatase, esterase (C4), esterase lipase (C8), leucine arylamidase, valine arylamidase, acid phosphatase, and naphthol-AS-BI-phosphohydrolase. In addition, API 20NE tests show positive results for nitrate (NO_3_) to nitrite (NO_2_^-^) reduction and esculin hydrolysis. The G + C content is 69.9%. The GenBank/EMBL/DDBJ accession numbers for the 16S rRNA gene sequence and the assembled genome sequence of strain HM2^T^ are MN498047 and JADDIV000000000, respectively.

The type strain HM2^T^ (= KCTC 82557^T^ = NBRC 114489^T^) was isolated from soil at Seopjikoji, Jeju Island, Republic of Korea.

## Supplementary Information

Supplementary Information.

## Data Availability

Generated sequences can be found as stated under the species description.
