# Supplementary material for: Comparative genomic analyses of four novel Ramlibacter species and the cellulose-degrading properties of Ramlibacter cellulosilyticus sp. nov
Source: Sci Rep. 2022 Dec 8;12:21233. doi: 10.1038/s41598-022-25718-w (PMC9731981; doi:10.1038/s41598-022-25718-w)

***Supplementary Data***

Comparative genomic analyses of four novel *Ramlibacter* species and the cellulose-degrading properties of *Ramlibacter cellulosilyticus* sp. nov.

**Table S1.** Fatty acid compositions of strains USB13^T^, AW1^T^, GTP1^T^, and HM2^T^ and their reference strains. Strains: 1, USB13^T^; 2, AW1^T^; 3, GTP1^T^; 4, HM2^T^; *R. monticola* KACC 19175^T^; 6, *R. alkalitolerans* KACC 19305^T^; 7, *R. ginsenosidimutans* KACC 17527^T^; 8, *R. humi* KCTC 52922^T^; 9, *R. henchirensis* KACC 11925^T^; 10, *R. tataouinensis* KACC 11924^T^; 11, *R. rhizophilus* KCTC 52083^T^. All data were obtained from this study. Fatty acids accounting for less than 1% are indicated as TR; if not detected, indicated as -. Therefore, the percentages may not add up to 100%.

| **Fatty Acids** | **1** | **2** | **3** | **4** | **5** | **6** | **7** | **8** | **9** | **10** | **11** |
| --- | --- | --- | --- | --- | --- | --- | --- | --- | --- | --- | --- |
| Saturated: |  |  |  |  |  |  |  |  |  |  |  |
| C_10:0_ | TR | TR | - | 3.0 | 1.0 | - | TR | - | TR | TR | TR |
| C_10:0_ iso | - | - | 3.5 | - | - | - | - | - | - | - | - |
| C_11:0_ | TR | TR | 4.6 | 1.5 | 2.1 | - | 2.3 | 1.1 | 1.5 | 1.1 | 2.2 |
| C_12:0_ | - | 7.8 | - | - | - | - | 1.9 | - | - | **14.7** | **16.6** |
| C_14:0_ | TR | TR | - | TR | 3.0 | 3.6 | 4.5 | - | 1.7 | TR | - |
| C_15:0_ | - | TR | 4.4 | 3.8 | 2.4 | 2.8 | 1.1 | 3.6 | 4.7 | 1.6 | 1.3 |
| C_15:0_ anteiso | 8.6 | TR | TR | TR | 1.0 | TR | TR | - | - | - | TR |
| C_16:0_ | **27.4** | **29.2** | **24.6** | **24.8** | **23.9** | **33.8** | **30.4** | **35.2** | **34.8** | **22.7** | **31.0** |
| C_17:0_ | TR | TR | 2.5 | 1.1 | 1.5 | TR | - | 1.7 | 1.5 | 1.6 | - |
| C_18:0_ | 5.8 | 3.2 | 6.9 | 3.0 | 5.9 | 5.6 | TR | 4.6 | 4.9 | 3.1 | 6.3 |
|  |  |  |  |  |  |  |  |  |  |  |  |
| Hydroxy: |  |  |  |  |  |  |  |  |  |  |  |
| C_8:0_ 3-OH | TR | 1.0 | - | TR | 1.0 | - | - | 2.8 | 3.6 | 3.3 | 2.0 |
| C_9:0_ 3-OH | TR | - | - | TR | 1.1 | - | - | 1.6 | 1.2 | TR | - |
| C_10:0_ 3-OH | **11.6** | TR | - | 7.2 | **20.0** | - | 5.1 | 4.5 | - | 4.3 | 5.0 |
| C_11:0_ 3-OH | - | TR | 1.5 | - | TR | 1.9 | TR | - | - | - | - |
|  |  |  |  |  |  |  |  |  |  |  |  |
| Unsaturated: |  |  |  |  |  |  |  |  |  |  |  |
| C_14:1_ ω5c | TR | TR | 1.1 | TR | TR | 1.1 | TR | - | - | TR | TR |
| C_15:1_ ω6c | TR | TR | 3.8 | 1.0 | 1.2 | 1.8 | TR | TR | TR | TR | - |
| C_17:0_ cyclo | 3.4 | **22** | - | **27.4** | **10.7** | **11.5** | **29.6** | **27.7** | **25.0** | **18.2** | **21.5** |
| C_17:1_ ω6c | - | - | 3.7 | - | - | - | - | - | - | - | - |
|  |  |  |  |  |  |  |  |  |  |  |  |
| Summed Features*: |  |  |  |  |  |  |  |  |  |  |  |
| 2 | - | TR | - | - | 1 | - | - | - | TR | 2.3 | - |
| 3 | **28.6** | **14.1** | **30.3** | **11.2** | **14.1** | **24.0** | **17.2** | 5.7 | **10.6** | **12.7** | 5.5 |
| 7 | - | - | 1.7 | - | - | - | - | - | - | - | - |
| 8 | 7.2 | **10.0** | 2.5 | **10.6** | 5.3 | 4.5 | 2.9 | 5.2 | 4.3 | 7.7 | 4.3 |

* Summed features represent groups of two or three fatty acids that cannot be separated using the MIDI system.

Summed feature 2 (comprising C_14:0_3-OH and/or iso-C_16:1_ I)

Summed feature 3 (comprising C_16:1_ ω7c and/or C_16:1_ ω6c)

Summed feature 7 (comprising C_19:0_ cyclo ω10c and/or C_19:1_ω6c)

Summed feature 8 (comprising C_18:1_ ω7c and/or C_18:1_ ω6c)

**Figure S1.** Two-dimensional thin-layer chromatography (TLC) polar lipid profiles of novel strains (A), USB13^T^; (B), AW1^T^; (C), GTP1^T^; (D), HM2^T^. Lipids were visualized by spraying plates with 5% molybdophosphoric acid followed by 5 min of heating at 110 °C. Abbreviations: DPG, diphosphatidylglycerol; GL, unidentified glycolipid; L, unidentified polar lipid; PAL, identified phosphoaminolipid; PE, phosphatidylethanolamine; PG, phosphatidylglycerol; PGAL, unidentified phosphoglycoaminolipid; PGL, unidentified phosphoglycolipid; PL, unidentified phospholipid.


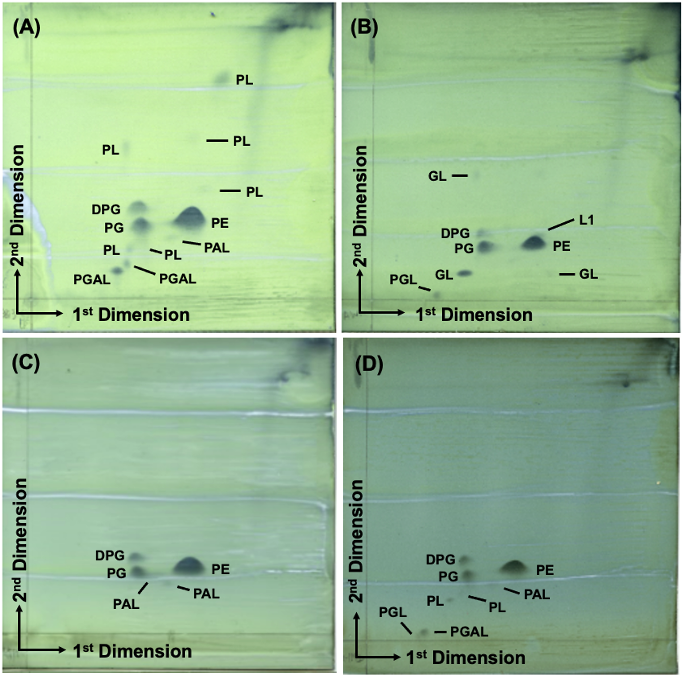


**Figure S2.** Transmission electron microscopy images of novel strains (A), USB13^T^; (B), AW1^T^; (C), GTP1^T^; (D), HM2^T^ grown in R2A broth for 3 days at 30 °C. Strains were negatively stained with 2% uranyl acetate for 2 sec. Bar, 0.2 μm.


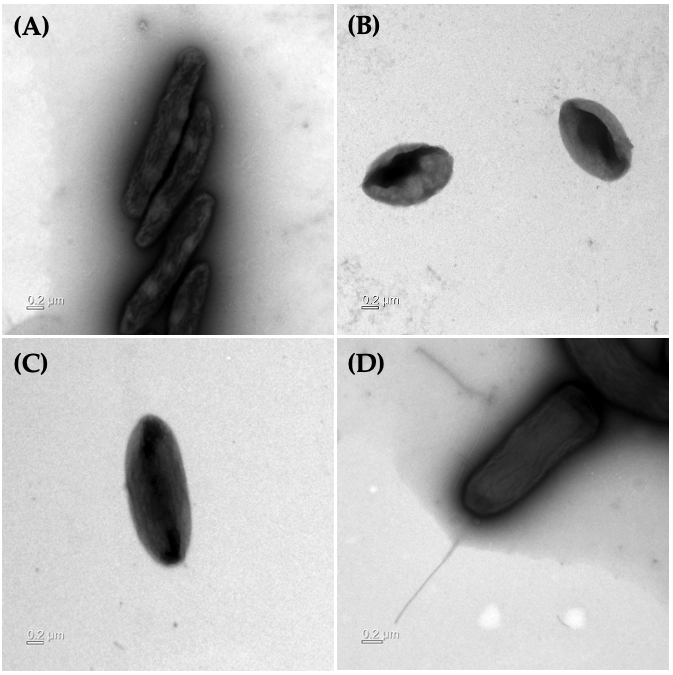


**Figure S3.** Maximum-likelihood (ML) tree of strains USB13^T^, AW1^T^, GTP1^T^, and HM2^T^ and closely related type strains. The phylogenetic tree was reconstructed based on 16S rRNA gene sequences with bootstrap values based on 1000 replications are listed as percentages at branching points. Only bootstrap values exceeding 50% are shown. The dots indicate nodes that were also recovered in the neighbor-joining and maximum-parsimony trees. Bar, 0.050 substitutions per nucleotide position.


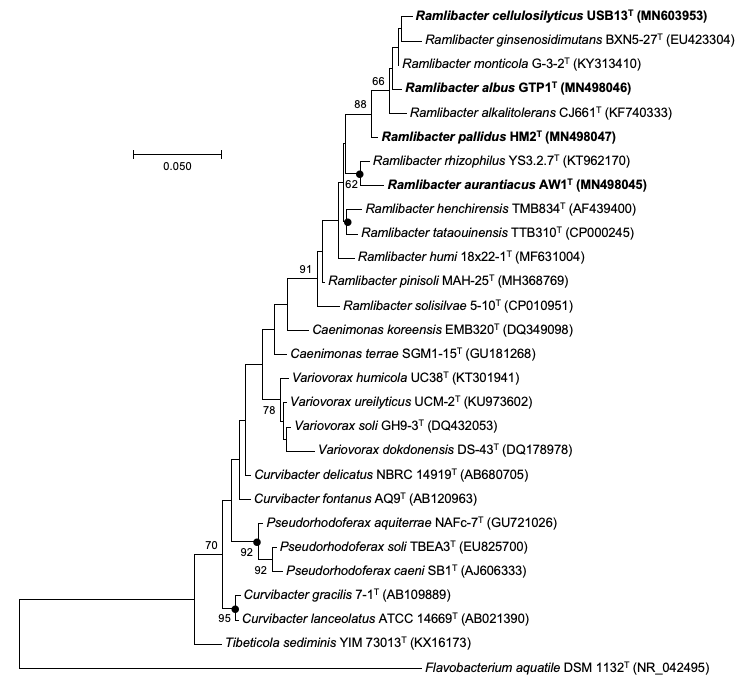


**Table S2.** Genomic features of the novel strains and closely related strains of *Ramlibacter*. Data for all strains were obtained from NCBI. Strains: 1, USB13^T^; 2, AW1^T^; 3, GTP1^T^; 4, HM2^T^; 5, *R. monticola* KACC 19175^T^; 6, *R. alkalitolerans* KACC 19305^T^; 7, *R. ginsenosidimutans* KACC 17527^T^; 8, *R. humi* 18x22-1^T^; 9, *R. henchirensis* DSM 14656^T^; 10, *R. tataouinensis* TTB310^T^; 11, *R. rhizophilus* CCTCC AB2015357^T^.

| **Features** | **1** | **2** | **3** | **4** | **5** | **6** | **7** | **8** | **9** | **10** | **11** |
| --- | --- | --- | --- | --- | --- | --- | --- | --- | --- | --- | --- |
| Assembled contigs | 34 | 32 | 39 | 7 | 20 | 33 | 26 | 24 | 6 | 1 | 152 |
| Genome Length (Mbp) | 5.53 | 5.11 | 6.15 | 4.31 | 6.14 | 5.71 | 5.33 | 4.47 | 4.79 | 4.07 | 4.59 |
| N50 length (bp) | 325,634 | 375,677 | 445,396 | 752,961 | 644,063 | 346,469 | 395,044 | 737,131 | 2,576,095 | N/A | 622,312 |
| G+C content (%) | 69.7 | 68.6 | 67.9 | 69.9 | 69.3 | 69.2 | 68.7 | 68.9 | 68.5 | 70.0 | 69.7 |
| Number of rRNAs | 7 | 3 | 3 | 6 | 6 | 3 | 6 | 3 | 3 | 3 | 3 |
| Number of tRNAs | 48 | 47 | 43 | 40 | 50 | 58 | 48 | 45 | 44 | 43 | 46 |
| Gene count | 5,195 | 4,859 | 6,004 | 4,282 | 5,908 | 5,501 | 5,170 | 4,269 | 4,643 | 3,991 | 4,377 |
| Protein count | 5,106 | 4,749 | 5,915 | 4,195 | 5,798 | 5,398 | 5,053 | 4,174 | 4,547 | 3,908 | 4,269 |

**Table S3.** List of CAZymes within the genomes of strains USB13^T^, AW1^T^, GTP1^T^, and HM2^T^ based on NCBI PGAP and CAZy database results.

| **Strains** | **GenBank  Accession No.** | **Start** | **Stop** | **Length** | **Protein Name** |
| --- | --- | --- | --- | --- | --- |
| USB13^T^ | MBC5782872.1 | 443959 | 445764 | 601 | glycoside hydrolase family 15 protein |
|  | MBC5784046.1 | 1 | 1358 | 611 | glycoside hydrolase family 15 protein |
|  | MBC5786150.1 | 37522 | 38472 | 316 | glycosyl hydrolase |
|  | MBC5786405.1 | 242622 | 244457 | 452 | glycoside hydrolase family 99-like domain-containing protein |
| AW1^T^ | MBL0419336.1 | 684255 | 686135 | 626 | glycoside hydrolase family 15 protein |
|  | MBL0419348.1 | 700466 | 702949 | 827 | glycoside hydrolase family 2 protein |
|  | MBL0419360.1 | 718342 | 719532 | 396 | glycoside hydrolase |
|  | MBL0419729.1 | 1117550 | 1119496 | 648 | glycoside hydrolase family 2 |
|  | MBL0420670.1 | 445006 | 446892 | 628 | glycoside hydrolase family 15 protein |
|  | MBL0421736.1 | 323749 | 326262 | 837 | cellulase family glycosylhydrolase |
|  | MBL0422555.1 | 173520 | 175340 | 606 | glycoside hydrolase family 15 protein |
|  | MBL0422926.1 | 126650 | 127747 | 365 | glycoside hydrolase family 5 protein |
| GTP1^T^ | MBC5763547.1 | 789048 | 790844 | 598 | glycoside hydrolase family 15 protein |
|  | MBC5764218.1 | 72101 | 73063 | 320 | family 16 glycosylhydrolase |
| HM1^T^ | MBE7366483.1 | 554877 | 556682 | 601 | glycoside hydrolase family 2 |
|  | MBE7367336.1 | 459626 | 461425 | 599 | glycoside hydrolase family 15 protein |
|  | MBE7368447.1 | 770418 | 772061 | 547 | glycoside hydrolase family 18 protein |

**Table S4.** Comparison of total number of CAZymes within the genomes of strains USB13^T^, AW1^T^, GTP1^T^, and HM2^T^.

| **CAZymes** | **USB13^T^** | **AW1^T^** | **GTP1^T^** | **HM2^T^** |
| --- | --- | --- | --- | --- |
| GH2 | 0 | 2 | 0 | 1 |
| GH5 | 0 | 1 | 0 | 0 |
| GH15 | 2 | 3 | 1 | 1 |
| GH16 | 0 | 0 | 1 | 0 |
| GH18 | 0 | 0 | 0 | 1 |
| GH99 | 1 | 0 | 0 | 0 |
| Unnamed GH | 1 | 2 | 0 | 0 |
| **Total CAZymes** | **4** | **8** | **2** | **3** |

**Table S5.** Comparison of COG gene count distribution of strains USB13^T^, AW1^T^, GTP1^T^, and HM2^T^.

| **Functional Groups** | **USB13^T^** | **AW1^T^** | **GTP1^T^** | **HM2^T^** |
| --- | --- | --- | --- | --- |
| **INFORMATION STORAGE AND PROCESSING** |  |  |  |  |
| [J] Translation, ribosomal structure, and biogenesis | 233 | 222 | 254 | 228 |
| [A] RNA processing and modification | 1 | 1 | 1 | 1 |
| [K] Transcription | 244 | 265 | 306 | 229 |
| [L] Replication, recombination, and repair | 129 | 128 | 129 | 115 |
| [B] Chromatin structure and dynamics | 5 | 3 | 4 | 4 |
| **CELLULAR PROCESSES AND SIGNALING** |  |  |  |  |
| [D] Cell cycle control, cell division, and chromosome partitioning | 42 | 42 | 39 | 30 |
| [Y] Nuclear structure | 0 | 0 | 0 | 0 |
| [V] Defense mechanisms | 72 | 65 | 97 | 69 |
| [T] Signal transduction mechanisms | 294 | 214 | 349 | 264 |
| [M] Cell wall/membrane/envelope biogenesis | 248 | 218 | 234 | 192 |
| [N] Cell motility | 103 | 88 | 62 | 83 |
| [Z] Cytoskeleton | 3 | 3 | 4 | 2 |
| [W] Extracellular structures | 0 | 1 | 2 | 0 |
| [U] Intracellular trafficking, secretion, and vesicular transport | 43 | 60 | 66 | 42 |
| [O] Posttranslational modification, protein turnover, chaperones | 184 | 148 | 203 | 159 |
| **METABOLISM** |  |  |  |  |
| [C] Energy production and conversion | 421 | 448 | 473 | 269 |
| [G] Carbohydrate transport and metabolism | 258 | 181 | 230 | 212 |
| [E] Amino acid transport and metabolism | 380 | 294 | 446 | 329 |
| [F] Nucleotide transport and metabolism | 78 | 69 | 77 | 72 |
| [H] Coenzyme transport and metabolism | 193 | 219 | 221 | 162 |
| [I] Lipid transport and metabolism | 274 | 275 | 437 | 235 |
| [P] Inorganic ion transport and metabolism | 179 | 246 | 221 | 161 |
| [Q] Secondary metabolites biosynthesis, transport, and catabolism | 107 | 161 | 140 | 96 |
| **POORLY CHARACTERIZED** |  |  |  |  |
| [R] General function prediction only | 281 | 247 | 387 | 237 |
| [S] Function unknown | 239 | 188 | 227 | 219 |
| [X] Assigned multiple COG | 31 | 32 | 45 | 2 |
| **Total gene count** | **4042** | **3818** | **4654** | **3412** |

**Figure S4.** Circular representation of whole genome sequences strains USB13^T^, AW1^T^, GTP1^T^, and HM2^T^. Strain USB13^T^ was set as the reference genome. Rings (from inner to outer): 1, GC content; 2, GC skew; 3, WGS (Whole Genome Sequence) of strain USB13^T^ (reference); 4, WGS strain AW1^T^; 5, WGS of strain GTP1^T^; 6, WGS of HM2^T^. Genome map created using BRIG platform application^38^.


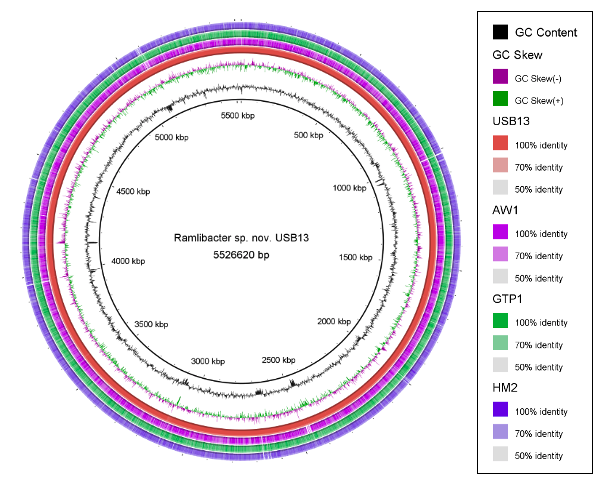

Supplement: Supplementary file 1 — Supplementary Information. [file 41598_2022_25718_MOESM1_ESM.docx]
